# Identification of Neurodegenerative Diseases Based on Vertical Ground Reaction Force Classification Using Time–Frequency Spectrogram and Deep Learning Neural Network Features

**DOI:** 10.3390/brainsci11070902

**Published:** 2021-07-08

**Authors:** Febryan Setiawan, Che-Wei Lin

**Affiliations:** 1 Department of Biomedical Engineering, College of Engineering, National Cheng Kung University, Tainan 701, Taiwan; febryans2802.wtmh@gmail.com; 2 Medical Device Innovation Center, National Cheng Kung University, Tainan 701, Taiwan

**Keywords:** gait analysis, neuro-degenerative diseases, time–frequency spectrogram, deep learning, vertical ground reaction force signal

## Abstract

A novel identification algorithm using a deep learning approach was developed in this study to classify neurodegenerative diseases (NDDs) based on the vertical ground reaction force (vGRF) signal. The irregularity of NDD vGRF signals caused by gait abnormalities can indicate different force pattern variations compared to a healthy control (HC). The main purpose of this research is to help physicians in the early detection of NDDs, efficient treatment planning, and monitoring of disease progression. The detection algorithm comprises a preprocessing process, a feature transformation process, and a classification process. In the preprocessing process, the five-minute vertical ground reaction force signal was divided into 10, 30, and 60 s successive time windows. In the feature transformation process, the time–domain vGRF signal was modified into a time–frequency spectrogram using a continuous wavelet transform (CWT). Then, feature enhancement with principal component analysis (PCA) was utilized. Finally, a convolutional neural network, as a deep learning classifier, was employed in the classification process of the proposed detection algorithm and evaluated using leave-one-out cross-validation (LOOCV) and *k*-fold cross-validation (*k*-fold CV, *k* = 5). The proposed detection algorithm can effectively differentiate gait patterns based on a time–frequency spectrogram of a vGRF signal between HC subjects and patients with neurodegenerative diseases.

## 1. Introduction

Amyotrophic lateral sclerosis (ALS), Huntington’s disease (HD), and Parkinson’s disease (PD), as NDDs, are defined as diseases caused by the progressive death of neurons in different regions of the nervous system, through the loss of structure and function of neurons [1]. For example, PD is the second most prevalent NDD, with a prevalence of 0.3% in the general population, ~1% in the elderly over 60 years old, and ~3% in those aged 80 years old or more [2]. PD incidence rate ranges between 8 and 18 people out of 100,000 per year [2]. The median age at onset is 60 years, and the average time it takes for the disease to progress, from the diagnosis to death, is approximately 15 years [2]. Men show a 1.5–2 times greater prevalence of this disease and incidence compared to women [2]. In terms of the medication required for treatment, PD costs USD 2500 each year, and therapeutic surgery costs up to USD 100,000 per patient [3]. ALS is the third most prevalent NDD and the most common motor neuron disease, with an estimated annual incidence of 1.9 people out of 100,000 per year [4,5]. In the United States, 30,000 people have ALS, 30,000 have HD, and 1 million have PD [6]. As NDDs mainly affect people in their middle to late years of life, the incidence is expected to increase with the an increasingly aging population. In 2030, 1 out of every 5 Americans will be over the age of 65, and 30 years from now, more than 12 million Americans will be affected by NDDs [7]. The development of early detection, treatments, and cures for NDDs is an ultimate goal of increasing urgency. NDDs can affect a variety of bodily functions, including heart rate, respiration, speech, mental function, balance, and movement. As the central nervous system, particularly the basal ganglia, controls the general motion (flexion and extension) of lower limbs, the gait of a patient with an NDD will become abnormal (different gait pattern than a healthy subject) due to motor neuron decline [8]. ALS, also known as motor neuron disease (MND), is characterized by stiff muscles, muscle twitching, and steadily deteriorating weakness as muscles decrease in size [9,10,11]. HD is a hereditary disorder that causes the death of brain cells, resulting in a lack of coordination, a shaky gait, and uncoordinated and jerky body movements as the disease progresses [12,13,14]. PD is a long-term degenerative disorder of the central nervous system that primarily affects the motor system. Early symptoms include trembling, rigidity, slowness of movement, and difficulty walking [15,16,17]. It is reasonable to assume that these NDDs have an impact on foot force as a result of this phenomenon. For movement analyses in HC subjects and other subjects with various diseases, gait data have been developed. This type of approach is very useful for understanding the movement disorder in NDDs and has a large amount of potential in terms of presenting non-invasive automatic NDD classification methods.

Gait analysis research has been developed in the last decade, particularly using time series of stride, stance or swing intervals, ground reaction force (GRF), and foot force. Previous research has shown that feature extraction methods and machine learning can be used to classify gait features. Xia et al. suggested a method for classifying gait rhythm signals in patients with NDDs and healthy people [18]. They tested various classification models and statistical characteristics, such as a support vector machine (SVM), random forest (RF), multi-layer perceptron (MLP), and k-nearest neighbor (KNN). To perform feature extraction of PD subjects based on sensor signals, Ertugrul et al. developed shifted one-dimensional local binary patterns and used Bayes network (BayesNT), naive Bayes (NB), logistic regression (LR), partial C4.5 decision tree (PART), a rule learner approach (Jrip), functional tree (FT), and other classifiers. [19]. Using entropy parameters, Wu et al. measured signal fluctuations in gait rhythm time series of PD patients [20]. Their research aimed to calculate the approximate entropy (ApEn), normalized symbolic entropy (NSE), and signal turns count (STC) parameters for measuring stride fluctuations in PD. Nonlinear gait pattern classifications were performed using generalized linear regression analysis (GLRA) and support vector machines (SVM). Bilgin investigated the effect of feature extraction on ALS patient classification in NDD and HC subjects [21]. The input signal, compound foot (CF) force, was decoded for feature extraction using a 6-level discrete wavelet transform (DWT) with several wavelet methods. In linear discriminant analysis (LDA) and naive Bayesian classifier (NBC), the derived features were validated using 20 trials for 5-fold cross-validation.

Deep learning has demonstrated excellent performance in gait classification problems in recent years. Zeng and Wang, for example, introduced a technique based on gait dynamics to classify (diagnose) NDDs using deterministic learning theory and a recurrent neural network (RNN) [22]. Zhao et al. used dual-channel long short-term memory (LSTM)-based multi-feature extraction on gait for NDD diagnosis [23]. They developed a dual-channel LSTM model to combine gait time series and force series recorded from NDD patients in order to understand the whole gait. According to several electrocardiogram (ECG) classification studies [24,25,26,27], combining a time–frequency representation (spectrogram) with a deep neural network can improve performance in extracting the distribution of important features more easily and automatically learn complex representation features directly from data. The goal of feature enrichment in a spectrogram of a time series signal is to enrich the simplified representation by restoring topological information, neighborhood information, and association information with details [28]. A deep neural network, on the other hand, has excellent feature extraction capabilities and can automatically extract “deep features” [29], greatly improving classification accuracy.

The specific aim of this study was to observe the effectiveness of the utilization of several feature transformations from a 1D vGRF signal into a 2D time–frequency spectrogram and the combination of principal component analysis (PCA) with a deep learning network for extracting features for the classification of NDD patients. The technological impact of this paper is that it is the first to apply spectrogram- and deep learning-based networks to the gait classification problem and yield high classification accuracy. The emphasis of this paper is on gaining insight into the effectiveness of the left foot (LF), right foot (RF), and compound the foot (CF) force signals in the classification of NDDs. It warrants investigation into whether the three types of degenerative nerve diseases (ALS, HD, and PD) interfere with a patient’s ability to handle two-foot propulsion and if the major difference in vGRF is related to the type of disease the patient has.

Raw vGRF signal data from NDD and HC subjects were obtained as the system’s input using force-sensitive resistors, with the output approximately proportional to the force under the foot [30]. Continuous wavelet transform (CWT), short-time Fourier transform (STFT), and wavelet synchrosqueezed transform (WSST) feature transformations were applied to the input in order to create new features (time–frequency spectrogram) from existing ones. Then, to increase classification performance, principal component analysis (PCA) was applied to the time–frequency spectrogram by selecting the features’ principal components (PCs). Training and testing sets were created for the PCs of HC and NDD subjects. Several classification parameters were created by training the estimators on the training sets and comparing them to a test set of the HC or NDD to be categorized. In this study, a convolutional neural network (CNN) was successfully used to classify the HC and NDD in the classification stage (training and testing phase). The proposed method can effectively distinguish between HC and NDD gait patterns.

## 2. Materials and Methods

**Figure 1:**
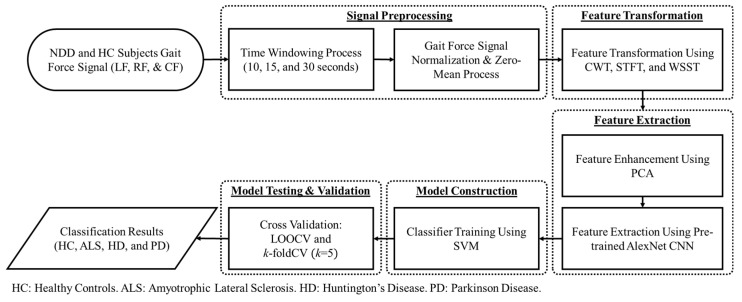
Flowchart of the proposed NDD detection algorithm using CWT as the feature transformation.

By transforming one-dimensional signals into two-dimensional pattern objects (images) using the feature transformation technique from a continuous wavelet transform (CWT), the proposed NDD detection algorithm attempted to extract pattern characteristics and visualization from vGRF signals in ALS, HD, PD, and HC subjects. The proposed NDD detection algorithm consists of four main steps, as shown in Figure 1: (1) signal preprocessing of NDD and the HC vGRF signal; (2) feature extraction by generating the spectrogram of the vGRF signal using CWT and PCA; (3) construction of the classifier model by feature training using Pretrained AlexNet CNN; and (4) the use of cross-validation techniques to test and analyze the effectiveness of the detection algorithm based on the classifier model.

### 2.1. Neuro-Degenerative Diseases Gait Dynamics Database

Hausdorff et al. presented the vGRF database used in this study (called the Gait Dynamics in Neuro-Degenerative Disease Database) online in the PhysioNet database [31]. This database’s raw signal data were obtained by using force-sensitive resistors with an output proportional to the force under the foot. When loaded, the transducer was a conductive polymer layer sensor with a changed resistance. The sensor was chosen due to its 0.05-inch thickness, temperature insensitivity, rapid dynamic response, ability to restrain an overload, and electronically simple interface. Two 1.5-in^2^ force-sensitive resistors were used, and the sensors were taped to an insole that was used to position them inside the shoe. The insole was made by tracing an outline of the foot onto the manila folder and then cutting out the tracing. One sensor was placed near the toes and metatarsals in the anterior part of the insole, and the other was placed near the heel on the opposite end. The two footswitches were connected in parallel and functioned as a single large sensor (the output from these two footswitches were added up). To increase the signal saturation, a 390 resistor, R1, was placed in series with this parallel connection as a voltage divider. A 5-V battery-operated circuit powered the sensors. The divider’s output voltage was fed into a voltage follower, of which the voltage output nonlinearly increased as the force was increased. The switch’s output voltage ranges from 0 V with no load to 3.5 V with a full load (closed). The analog signal was then converted into digital format and analyzed with software [30].

There are 64 recordings of information from 13 patients with ALS, 20 patients with HD, 15 patients with PD, and 16 healthy controls in the database. This database contains two types of data: raw force series data and derived time series from the raw data. The force series comprises LF force and RF force signal. Left stride interval (s), right stride interval (s), left swing interval (s), right swing interval (s), left swing interval (% of stride), right swing interval (% of stride), left stance interval (s), right stance interval (s), left stance interval (% of stride), right stance interval (% of stride), double support interval (s), and double support interval (% of stride) are contained within the time-series data.

### 2.2. Signal Preprocessing

During the data collection, a 5-min vGRF signal was obtained. The proposed technique took three types of vGRF signals as input: LF, RF, and CF (CF = LF + RF). Due to the length of the foot force signal, it was difficult to interpret the data even after using a CWT to transform the features. The window function, a mathematical term that is zero-valued outside of a specified interval, was used to visualize the foot force signal clearly. The time windows used in this study were 10, 30, and 60 s. The time windowing determination was helpful in obtaining more data to feed into the deep learning model and simulating more precise and fast disease predictions [32].

### 2.3. Feature Transformation

#### 2.3.1. Continuous Wavelet Transform (CWT)

A continuous wavelet transform (CWT) is a signal processing technique for observing nonstationary signals’ time-varying frequency spectrum characteristics [33]. The CWT result is a time–frequency spectrogram (time–scale representation), which provides useful information on the relationship between time and frequency.

The CWT of a time series function $x\left(t\right)\in L^{2}\left(\mathbb{R}\right)$ with a scaling factor $s\in {\mathbb{R}}^{+}\;(s>0)$ that controls the wavelet’s width and a translation parameter τ controls the wavelet’s location can be expressed by the following equation:

$$X_{w}\left(s,\tau \right)=\frac{1}{\sqrt{s}}\overset{\infty }{\underset{-\infty }{\int }}x\left(t\right)\psi^{*}\left(\frac{t-\tau }{s}\right)\;dt$$

where *ψ*(*t*) is a mother wavelet, also called a window function. A Morlet or Gabor wavelet was used as the mother wavelet function in this study. This wavelet function is made up of a complex sinusoid with a Gaussian window (a complex exponential multiplied by a Gaussian window) that is specified by the following term:

$$\psi_{\omega_{0}}\left(t\right)=\left(e^{-ift}-e^{-\frac{1}{2}f^{2}}\right)e^{-\frac{1}{2}t^{2}}$$

Parameter t refers to the time and f represents the reference frequency.

**Figure 2:**
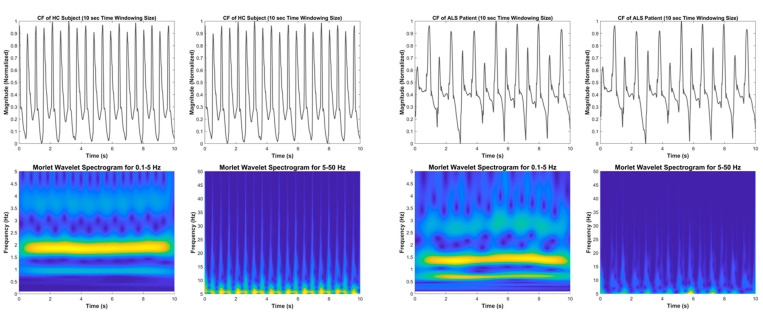
Time–frequency spectrogram using the CWT of the CF vGRF signal of HC (**left**) and ALS (**right**) subjects in 10-s time windows.

**Figure 3:**
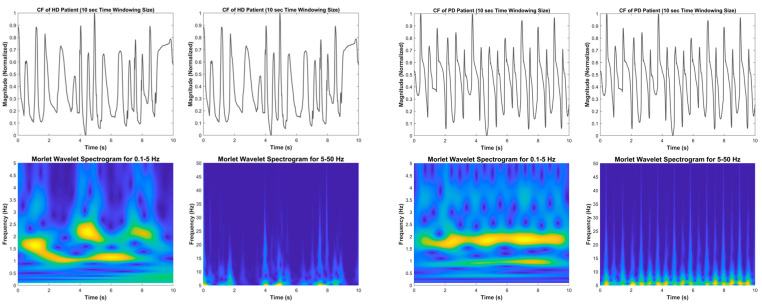
Time–frequency spectrogram using the CWT of the CF vGRF signal of HD (**left**) and PD (**right**) subjects in 10-s time windows.

The vGRF signal is represented as a time–frequency spectrogram image by the time–frequency transformation applied to the system. The image clearly shows distinct vGRF patterns for HC and NDD subjects that are not visible in the signal’s time and frequency domains. Variations in the foot pressure signal caused by temporal characteristics can also be studied using the time–frequency spectrogram. The measurement of step length, stance width, the length of the step rhythm, and step velocity are all examples of temporal characteristics, which are also known as spatial characteristics or linear gait variabilities. The CWT feature transformation results for the NDD and HC groups are shown in Figure 2 and Figure 3. 

#### 2.3.2. Short Time Fourier Transform

The short-time Fourier transform (STFT) is a series of Fourier-related transforms applied to a windowed signal to determine the sinusoidal frequency and phase content of local parts as the signal transforms over time [34]. *STFT* is calculated by dividing a longer time signal into shorter segments of equal lengths and then computing the Fourier transform on each shorter segment independently.

The *STFT* pair is given as follows:

$$\{\begin{array}{c} X_{STFT}\left[m,n\right]=\overset{L-1}{\underset{k=0}{\sum }}x\left[k\right]g\left[k-m\right]e^{-j2\pi nk/L}\\ x\left[k\right]=\underset{m}{\sum }\underset{n}{\sum }X_{STFT}\left[m,n\right]g\left[k-m\right]e^{j2\pi nk/L} \end{array}$$

where *x*[*k*] represents a signal and *g*[*k*] represents an L-point window function. The *STFT* of *x*[*k*] can be construed as the Fourier transform of the product *x*[*k*]*g*[*k* − *m*].

#### 2.3.3. Wavelet Synchrosqueezed Transform (WSST)

The wavelet synchrosqueezed transform is a time–frequency analysis technique for studying multi-component signals with oscillating modes (speech waveforms, machine vibrations, and physiologic signals), with the goal of sharpening a time–frequency analysis by reallocating the signal energy in frequency [35]. The synchrosqueezing algorithm uses CWT of the input signal to generate the instantaneous frequency information. The instantaneous frequencies from the CWT output, $W_{f}$, are extracted using a phase transform, $\omega_{f}$. This phase transform is proportionate to the first derivative of the CWT with respect to the translation, u.

$$\omega_{f}\left(s,u\right)=\frac{\partial tW_{f}\left(s,u\right)}{2\pi iW_{f}\left(s,u\right)}$$

s are the scales, defined as $s=\frac{f_{x}}{f}$, where $f_{x}$ is the peak frequency and f is the frequency. Finally, “squeeze” the CWT over regions where the phase transformation is constant. The resulting instantaneous frequency value is redefined to a single value at the centroid of the CWT time–frequency region.

### 2.4. Principal Component Analysis (PCA) for Feature Enhancement

**Figure 4:**
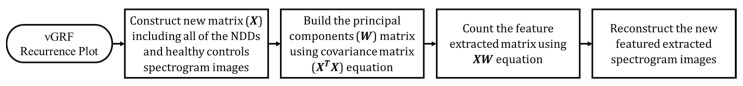
Flowchart of new feature extracted reconstruction using principal component analysis (PCA) as feature enhancement purpose.

The main idea behind a principal component analysis (PCA) is to reduce the dimension of a dataset with a large number of interrelated variables while minimizing the amount of variance in the dataset [36,37,38]. Specifically, PCA is able to minimize input data redundancy, remove potential association, and extract the most important feature vectors for data changing directions. This is accomplished by converting the dataset into a new set of variables known as principal components (PCs), which contain decorrelated and ordered variables. The PCA technique was mathematically characterized in this study using the steps below (as shown in Figure 4).

The aim of using PCA as a feature enhancement in this study was to improve between-class separability while reducing within-class separability [32]. Its goal was to increase the performance of deep learning in extracting features and artificial intelligence in classifying data points into the correct groups. In a deep learning network, such as CNN, the gradient diffusion problem occurs [39,40], and many of the filters in the layer are highly correlated thus making it possible to detect the same feature [41], and making insignificant contributions to the classification accuracy performance. To alleviate these problems by initializing the weights of convolution kernels, PCA is employed to the unsupervised extraction of input image eigenvectors [39,41,42]. PCA can also improve the classification performance (accuracy, sensitivity, specificity, and AUC value; see Section 3 Experimental Results).

### 2.5. Pre-Trained Convolutional Neural Network (CNN) as Feature Extractor

As in a simple multilayer neural network (deep learning), a convolutional neural network (CNN) is made up of one or more convolutional layers (often with subsampling and pooling layers) followed by one or more fully connected layers [43]. The architecture of a CNN is designed to take advantage of the input’s 2D structure (image or signal). This is achieved using local connections and weights, which are then followed by any pooling function that produces translation-invariant features. A CNN also has the advantage of being easier to train and having fewer parameters than other fully connected networks with the same number of hidden layers. The use of a CNN in the proposed method is primarily to differentiate between the time–frequency spectrogram representation of vGRF from HC and NDD (ALS, HD, and PD) subjects.

The proposed method used a pre-trained AlexNet CNN from the MATLAB R2018a Deep Learning Toolbox™ (The MathWorks, Inc., Natick, MA, USA). There are 25 layers in the architecture, including an input layer, five convolution 2D layers, seven ReLU (activation function) layers, two cross-channel normalization layers, three max pooling 2D layers, three fully connected layers, two dropout layers (for regularization), a softmax layer (normalized exponential function), and an output layer. The time–frequency spectrogram figure of the vGRF signal yielded by the CWT is fed into the AlexNet CNN in the proposed procedure. By using the layer activations as features, the pre-trained AlexNet CNN was proposed as a feature extractor [32,44,45,46]. This is a simple, time-efficient strategy to use pre-trained networks that avoids the effort needed to train a full network. By employing this simple and fast methodology, the possibility of wearable device integration with the algorithm becomes more promising. The suggested technique used a support vector machine (SVM) for classification and used the second fully connected layer as the feature extractor [47,48] (the CNN architecture utilized in this study is described in Table 1). AlexNet CNN has been trained with numerous common images, such as cars, boats, planes, dogs, and cats, but it is also possible for the CNN to utilize the distinct properties of non-image data (1D signal)−computationally efficient and locally focused—by converting non-image data into an image, such as a binary image [49], spectrogram [29,50], recurrence plot [32], or Gramian Angular Summation Field (GASF) image [51].

### 2.6. Support Vector Machine (SVM) as Classifier

In this study, the NDD patients and HC subjects were automatically distinguished using a support vector machine (SVM) after being processed based on feature transformation and extraction. The aim of the SVM is to construct a hyperplane or set of hyperplanes in a high- or infinite-dimensional space, which can be used for classification, regression, or other tasks, such as outlier detection [55]. Specifically, the purpose of using SVM is to discover an optimal decision surface that splits the dataset into correct classes and has a maximum distance or margin among the classes.

### 2.7. Cross-Validation

Cross-validation is a statistical method for evaluating and comparing learning algorithms that divide data into two groups: one for learning or training a model (training set) and another for validating the model (testing or validation set) [56,57,58]. In order for each data point to be confirmed, the training and testing sets must cross over in consecutive rounds. There are two primary reasons to use cross-validation: first, one algorithm can be used to investigate the performance of the learned model from available data. Specifically, it is used to assess an algorithm’s generalizability. The second goal is to compare the performance of two or more different algorithms and determine which is most appropriate for the data or to compare the performance of two or more variants of the parameterized model. Leave-one-out cross-validation (LOOCV) and *k*-fold cross-validation (*k*-fold CV, *k* = 5) were the two cross-validation methods used in this study.

## 3. Experimental Results

**Figure 5:**
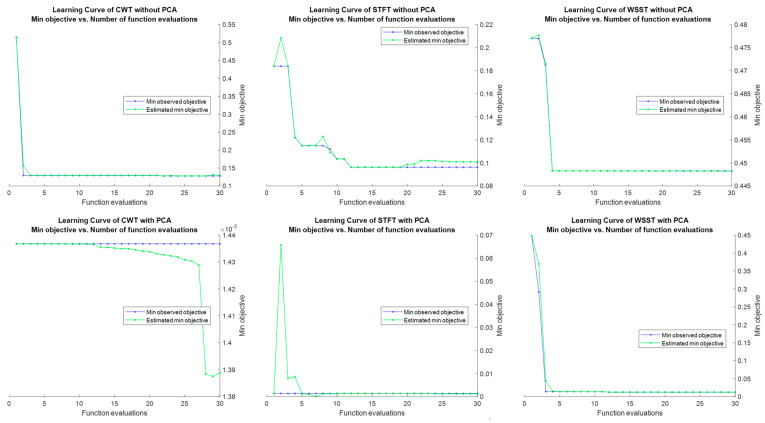
The learning curve of the SVM classifier with the feature extracted from fully connected layer of pre-trained AlexNet CNN as the input.

The experiments were run on an NVIDIA GeForce GTX 1060 6 GB computer with an Intel^®^ Core™ i5-8400 CPU @ 2.80 GHz, 2808 MHz, and 24 GB RAM, using MATLAB software (R2018a, The MathWorks, Inc., MA, USA). The number of time–frequency spectrogram images input (related to the time windowing process, where smaller time windowing results in more images and computation time becomes longer) and the number of neurons in the CNN corresponded to the calculation of the computation time (see Table 2). The proposed method’s accuracy, sensitivity, specificity, and ROC area under the curve (AUC) value were included as evaluation parameters, as specified in [59]. The learning curve contains the training loss function of the machine learning classifier, an SVM, as the result of feature extracted from fully connected layer of the pre-trained AlexNet CNN (see Figure 5).

When deciding between two or more diagnostic tests, Youden’s index is commonly used to assess the overall diagnostic test’s efficacy [60]. Youden’s index is a function of sensitivity and specificity that ranges from 0 to 1, with a value close to 1 indicating high diagnostic test effectiveness and that the test is perfect, and a value close to 0 indicating limited diagnostic test effectiveness and that the test is useless. The Youden’s index (J) is described as the sum of the two fractions representing the measurements properly diagnosed for the diseased (sensitivity) and HC (specificity) groups, overall cut-points c,−∞<c<∞:

$$J=\max_{c}\left\{\mathrm{sensitivity}\left(c\right)+\mathrm{specificity}\left(c\right)-1\right\}$$

### 3.1. Classification of the NDD and HC Group

In this classification scenario, there were three types of classification tasks: ALS versus HC, HD versus HC, and PD versus HC. In all classification scenarios, 13 ALS patients, 20 HD patients, 15 PD patients, and 16 HC subjects were used and observed, but the feedback signal for the proposed procedure was dependent on the time window in the time-windowing process and the frequency selection. There were 480 HC, 390 ALS, 600 HD, and 450 PD input signal numbers for the 10-s time window. The HC, ALS, HD, and PD input signal numbers were 160, 130, 200, and150, respectively, in the 30-s time window. There were 80, 65, 100, and 75 HC, ALS, HD, and PD input signal numbers in the 60-s time window, respectively. The detailed classification results are given in Table 3 and Table 4.

### 3.2. Classification among the NDD

In this study, a classification concepts were developed among the NDD, such as ALS vs. HD, PD vs. ALS, and HD vs. PD. The primary goal of this classification was to determine whether ALS, HD, and PD could be easily separated (the NDD group: ALS, HD, and PD). The conclusion was that the ALS group could easily be distinguished from the HD and PD groups, but that HD and PD were difficult to differentiate. In contrast to ALS vs. HD and PD vs. ALS, the HD vs. PD classification results were lower. This occurred due to the fact that both HD and PD are caused by basal ganglia degeneration, and the gait patterns of HD and PD patients are nearly identical [61]. The complete classification results are shown in Table 5 and Table 6.

### 3.3. Classification of All NDD in One Group with HC Group

The vGRF datasets of ALS, HD, and PD patients were merged into one group for NDD vs. HC classification, with the total number of NDD datasets varying depending on the time window. The experimental results for this classification situation are shown in Table 5 and Table 6.

### 3.4. Multi-Class Classification

As the physician may not know whether the patient is suffering from ALS, HD, or PD, the multi-class classification is closer to the clinical application. The entire vGRF dataset was divided into four categories based on disease patients (ALS, HD, and PD) and healthy subjects. For assessment and validation purposes, LOOCV and *k*-fold CV (*k* = 5) were also used in the multi-class classification. The detailed classification results are given in Table 7, Table 8 and Table 9.

## 4. Discussion

This section discusses the gait analysis of each NDD using the time and frequency analysis of the time–frequency spectrogram. Certain key features of a signal are difficult to notice with the naked eye, but time–frequency spectrogram analysis may aid in the discovery of significant time and frequency characteristics. The time–domain signal was transformed into the time–frequency domain using CWT in this research. Pattern visualization and recognition of the time–frequency spectrogram could easily be used to understand the NDD and HC gait phenomena.

This observation was limited to the CF vGRF signal. As this type of input signal is the extra force between the LF and RF force signals, it defines the correlations between the LF and RF features rather than each individual feature. As the input signal was shorter and the gait phenomenon could be studied in greater detail, a time window of 10 s was chosen. Based on the normal frequency of leg movements [62] and in order to obtain a high level of visualization, the frequency ranges of 0.1–5 Hz and 5–50 Hz were selected to observe the CWT time–frequency spectrogram in detail.

### 4.1. Healthy Control

The normal gait phenomenon was interpreted by observing the time–frequency spectrogram of the HC subject shown in Figure 2 (left). At the 0.1–5 Hz spectrogram, the strongest walking force magnitude (yellow) of the normal gait occurred at 1.6–2.1 Hz and was stable from the initial time until the end. This means that the foot force distribution and walking velocity of normal subjects are the same when they are walking. It was also shown that at 3 Hz and around 4.5–5 Hz, small areas, signifying the lowest force magnitude (dark blue), appeared alternately with a significant force magnitude (light blue) forming a regular pattern. This phenomenon appeared in the spectrogram caused by the CF force signal at the lowest magnitudes. There are three lowest magnitudes that can be observed in one cycle of the CF force time–domain signal (see Figure 2 (left) vGRF signal); each of these lowest magnitudes has an almost equal magnitude in every cycle of the signal. The lowest magnitudes (global minimum) that occurred at the beginning and end of the half gait cycle (only LF or RF gait cycle), close to the 0 force unit, show the toe-off and initial contact and the lowest magnitude (local minimum) that occurred in the half gait cycle exhibited when only one foot was in contact with the ground.

At the 5–50 Hz frequency range, there was also a steady, strong force level (yellow) of around 5 Hz, the same magnitude as that occurring during walking, from the initial to the end, and a significant force magnitude (light blue) still occurred up to 50 Hz and was also constant every time. Both time–frequency spectrograms indicate that the time and frequency components in the spectrogram comprise a regular pattern. This interpretation became a benchmark for the investigation into the NDD gait phenomenon. It was compared to analyze and discover the gait characteristics of NDDs based on the spectrogram.

### 4.2. Amyotrophic Lateral Sclerosis

For the ALS syndrome, as shown in Figure 2 (right), the most intense walking force attenuation of these patients in the 0.1–5 Hz spectrogram occurred at approximately 0.6–0.9 Hz and 1.1–1.5 Hz, which was lower than the frequency of the HC. This means that ALS patients walk in a more delayed fashion than the HC. The CF force time–domain signal shows that the lowest force magnitudes were not equal in every cycle of the CF force signal (clearly depicted in Figure 2 (right) vGRF signal); even at a specific time, the global minimum magnitudes were almost the same as the local minimum magnitudes and were not near the 0 force unit. In addition to these tendencies, there were more local minimum magnitudes along with the ALS time–domain signal. This phenomenon affects the regularity of the lowest force power pattern that typically occurs at 3 and 5 Hz. There were three frequency bands that showed the lowest force magnitude (dark blue), which appeared alternately with the significant force magnitude (light blue) forming an irregular pattern: at approximately 2–2.5 Hz, 3.5–4 Hz, and 5 Hz.

ALS patients had an unstable force magnitude (yellow) at 5 Hz, and at the 5–50 Hz frequency range, the instability only occurs at a specific time, at 6 and 7 s, and did not occur during the entire walking time. The significant force magnitude (light blue) was different every time and only reached 45 Hz.

### 4.3. Huntington’s Disease

Among the symptoms of HD are uncoordinated, jerky body movements that cause the patients to have severe gait abnormalities, especially in terms of their walking velocity. At specific times, it is faster than the HC, and at other times, it is slower. As shown in Figure 3 (left), on the 0.1–5 Hz spectrogram, the walking velocity of the HD patient arbitrarily changed corresponding with the time; for example, it can be seen that from the initial time to 2 s, the strongest force level (yellow) was at 1.5–2 Hz for 2 s until 4 s. The strongest force magnitude frequency decreased to 1 Hz, and there were two strong force magnitudes at 4 to 7 s, (1 Hz and 2–2.5 Hz). The CF force time–domain signal could not be distinguished between the global and local minimum as nearly all of the lowest force magnitudes were not close to the 0 force unit, which means at a specific time, both feet appeared to be in contact with the ground.

The 5–50 Hz spectrogram showed the strongest force power (yellow), where a significant force (light blue) only occurred at a specific period of time and had a different magnitude every time. Based on this observation, it can be concluded that the walking velocity of the HD subject fluctuated.

### 4.4. Parkinson’s Disease

As presented in Figure 3 (right), the time–frequency spectrogram of the PD subject is similar to that of the HC. The strongest force power was at 1.6–2 Hz and 1 Hz for the 0.1–5 Hz spectrogram and for the 5–50 Hz spectrogram, the strongest force magnitude (yellow) was approximately 5 Hz and the significant force power (light blue) occurred up to 50 Hz every time. However, the force magnitude was not distributed equally during the entire walking period. It was also obvious that the pattern of the lowest force magnitude was irregular at 2.5–5 Hz. This indicates that the global and local minimum magnitudes are not the same in every gait cycle. PD patients can exhibit walking velocity similar to that of a normal person, but their force distribution is typically not distributed equally due to the possibility of having a tremor.

### 4.5. Comparison Results with the Existing Literature

The comparison was made with the study by Zeng et al. [22]. The authors presented the gait dynamics method to classify NDD via the deterministic learning theory. They used LOOCV as the evaluation method only for ALS vs. HC, HD vs. HC, and PD vs. HC. They also employed an all-training-all-testing evaluation method for all their classification experiments, but in the current study, we did not use this method. A comparison was also made with the study by Zhao et al. [23]. They implemented dual-channel long short-term memory (LSTM)-based multi-feature extraction on gait for the diagnosis of NDD. Here, only accuracy results for ALS vs. HC, HD vs. HC, PD vs. HC, and NDD vs. HC were compared using LOOCV as the evaluation method.

We also compared our results with two studies from Tuan D. Pham [63], who proposed a novel method for gait analysis by transforming a time-series data sequence into images from which texture analysis methods and texture features of a gait can be extracted and presented the sensitivity, specificity, AUC value, and accuracy of HC vs. HD, HC vs. PD, and HC vs. ALS classifications using LOOCV as the evaluation method, and from Ren et al., who applied empirical mode decomposition in gait rhythm fluctuation analysis in neurodegenerative diseases subjects and used 10-fold cross-validation in order to overcome overfitting and obtained the AUC values of HD vs. HC, PD vs. HC, and ALS vs. HC [64]. A comparison of these studies with the results obtained using the proposed method is shown in Table 10.

In conclusion, the proposed method outperformed the classification results from Zeng et al., Zhao et al., and Ren et al. The NDD detection algorithm, proposed by Pham, obtained better results than the proposed method in the PD vs. HC classification. However, in the ALS vs. HC and HD vs. HC classifications, the proposed method achieved the same performance as the journal in terms of all evaluation parameters. However, the authors also used another method for classifying patients with PD (PD vs. HC) using linear discriminant analysis (LDA) and LOOCV was also performed as an evaluation technique with poor classification results. The accuracy only reached 77.42%.

## 5. Conclusions

This study used a time–frequency spectrogram based on a vGRF signal to implement a novel AI-based NDD detection algorithm. The ability to distinguish between the gait phenomena of NDD patients and a HC was achieved through pattern visualization and the recognition of the time–frequency spectrogram. By transforming the signal from the time–domain to the time–frequency domain, CWT was used to visualize the spectrogram of a gait foot force signal. By transforming the signal from the time–domain to the time–frequency domain, CWT was used to visualize the spectrogram of a gait foot force signal. To achieve good feature visualization, three-time window (10, 30, and 60 s) and three types of gait foot force signals were chosen as inputs (LF, RF, and CF force signals). Following the transformation of the original signal, feature enhancement using PCA was used to improve between-class separability while reducing within-class separability. Finally, CNN was used to classify the spectrogram images. Two types of cross-validation methods, LOOCV and *k*-fold CV (*k* = 5), were used to assess the CNN classification process, and four parameters were generated, including accuracy, sensitivity, specificity, and the AUC value. As a result, the proposed method outperformed state-of-the-art NDD detection methods in the literature for more than 95.32% of the parameters evaluated.

Despite the fact that the proposed method received importance-performance evidence, there are several significant areas in which it could be improved. First, since the proposed method was used to improve the performance of an existing database, clinical data should be obtained for verification and to address the database’s constraints (the limited number of NDD patients). Our own manufactured smart insole with an embedded 0.5” force-sensing resistor will be used to gather clinical data. Instead of walking down a long pathway, the NDD patient would be required to perform some basic daily tasks such as turning around and sitting. Second, long-term data collection for NDD progression is important for NDD patient therapy as the gait pattern of NDD patients should change over time as the disease progresses. Third, the NDD gait phenomenon based on a time–frequency spectrogram should be discussed with doctors to ensure the clinical meaning. Fourth, in order to validate and compare the efficiency of pattern visualization and recognition based on the use of a time–frequency spectrogram in NDD detection applications, other input data (such as kinetic data, temporal data, step length, and cadence) and classifiers should be used.

Based on pattern visualization and recognition using a deep learning classifier, the time–frequency spectrogram was successfully used to differentiate the gait phenomenon between NDD patients and a HC in this study. A fuzzy recurrence plot can also be used to implement and observe pattern visualization and recognition of the NDD gait phenomenon. A deep learning gait classification algorithmic based on fuzzy recurrence plot images could be used to improve NDD gait classification in the future.

## Figures and Tables

**Table 1:** CNN architecture of the proposed method.

Layer		Size	Hyperparameter	
			Weight	Bias
Input	Image	227×227×3	-	-
1	Convolution	55×55×96	11×11×3×96	1×1×96
2	ReLU ^1^	55×55×96	-	-
3	Cross Channel Normalization	55×55×96	-	-
4	Max Pooling ^2^	27×27×96	-	-
5	Grouped Convolution	27×27×256	5×5×48×128×2	1×1×128×2
6	ReLU ^1^	27×27×256	-	-
7	Cross Channel Normalization	27×27×256	-	-
8	Max Pooling ^2^	13×13×256	-	-
9	Convolution	13×13×384	3×3×256×3	1×1×384
10	ReLU ^1^	13×13×384	-	-
11	Grouped Convolution	13×13×384	3×3×192×192×2	1×1×192×2
12	ReLU ^1^	13×13×384	-	-
13	Grouped Convolution	13×13×256	3×3×192×128×2	1×1×128×2
14	ReLU ^1^	13×13×256	-	-
15	Max Pooling ^2^	6×6×256	-	-
16	Fully Connected	1×1×4096	4096×9216	4096×1
17	ReLU ^1^	1×1×4096	-	-
18	Dropout (50%) ^2^	1×1×4096	-	-
19	Fully Connected	1×1×4096	4096×4096	4096×1
20	SVM Classification Model ^3^	-	-	-
Output	Classification Output	-	-	-

^1^ Rectified linear unit as activation layer. ^2^ Max pooling and dropout layer can prevent the overfitting [52,53,54]. ^3^ The optimization hyperparameter of SVM is based on Bayesian optimization with 30 iterations.

**Table 2:** Average computation time of the proposed method.

Time Window	Total Number of vGRF Spectrogram	Elapsed Time (s)	
		LOOCV	*k*-Fold CV (*k* = 5)
10 s	1920	7933.684	33.013
30 s	640	873.829	12.708
60 s	320	235.986	7.473

**Table 3:** Summary results of two-class classification states (NDD and HC groups) using PCA.

Classification Task	Evaluation Parameter	Proposed Method					
		CWT + PCA (10 s/30 s/60 s)		STFT + PCA (10 s/30 s/60 s)		WSST + PCA (10 s/30 s/60 s)	
		LOOCV	*k*-Fold CV (*k* = 5)	LOOCV	*k*-Fold CV (*k* = 5)	LOOCV	*k*-Fold CV (*k* = 5)
ALS vs. HC	Sen (%)	100/100/100	100/**100**/**100**	100/100/100	100/**100**/**100**	**100**/100/100	100/100/100
	Spec (%)	99.79/99.38/98.77	99.79/**99.39**/**98.82**	99.79/99.38/98.77	99.79/**99.39**/**98.82**	**100**/98.77/98.77	99.79/98.79/97.65
	Acc (%)	99.89/99.66/99.31	99.89/**99.66**/**99.31**	99.89/99.66/99.31	99.89/**99.66**/**99.31**	**100**/99.31/99.31	99.89/99.31/98.62
	AUC	0.9990/0.9969/0.9938	1/**1**/**1**	0.9990/0.9969/0.9938	1/**1**/**1**	**1**/0.9938/0.9938	0.9987/0.9962/0.9846
HD vs. HC	Sen (%)	**100**/**100**/**100**	**100**/**100**/**100**	100/**100**/**100**	100/**100**/**100**	99.83/99.49/100	99.83/100/100
	Spec (%)	**100**/**100**/**100**	**100**/**100**/**100**	99.79/**100**/**100**	99.79/**100**/**100**	99.79/96.36/89.89	100/99.39/91.67
	Acc (%)	**100**/**100**/**100**	**100**/**100**/**100**	99.91/**100**/**100**	99.91/**100**/**100**	99.81/98.06/95	99.91/99.72/95.56
	AUC	**1**/**1**/**1**	**1**/**1**/**1**	0.9990/**1**/**1**	1/**1**/**1**	0.9981/0.9793/0.9494	1/1/1
PD vs. HC	Sen (%)	98.38/94.27/94.81	**99.13**/**97.47**/**100**	93.59/92.36/88.61	92.86/91.09/89.70	89.68/86.54/79.71	94.46/89.96/60.69
	Spec (%)	95.17/98.69/97.44	**94.53**/**96.42**/**96.47**	91.68/89.76/93.42	91.39/91.16/94.62	88.06/90.26/76.74	92.33/95.07/76.99
	Acc (%)	96.67/96.45/96.13	**96.45**/**96.77**/**98.06**	92.58/90.97/90.97	91.29/90.97/90.32	88.82/88.39/78.06	92.47/91.29/61.94
	AUC	0.9678/0.9648/0.9612	**0.9992**/**0.9969**/**0.9967**	0.9264/0.9106/0.9101	0.9794/0.9659/0.9679	0.8887/0.8840/0.7823	0.9957/0.9795/0.8763

Note: **bold and underlined** classification results were selected as the best based on Youden’s index.

**Table 4:** Comparison results of all two-class classification states between PCA and non-PCA (NDD and HC group) using *k*-fold CV (*k* = 5).

Classification Task	Evaluation Parameter	Proposed Method					
		CWT + PCA (10 s/30 s/60 s)		STFT + PCA (10 s/30 s/60 s)		WSST + PCA (10 s/30 s/60 s)	
		PCA	Non-PCA	PCA	Non-PCA	PCA	Non-PCA
ALS vs. HC	Sen (%)	**100**/**100**/**100**	94.97/96.26/89.02	**100**/**100**/**100**	87.88/88.49/93.14	**100**/**100**/**100**	75.69/76.30/86.11
	Spec (%)	**99.79**/**99.39**/**98.82**	92.73/95.23/94.21	**99.79**/**99.39**/**98.82**	93.74/93.65/91.78	**99.79**/**98.79**/**97.65**	65.49/77.41/62.59
	Acc (%)	**99.89**/**99.66**/**99.31**	93.56/95.52/91.72	**99.89**/**99.66**/**99.31**	90.69/91.03/91.03	**99.89**/**99.31**/**98.62**	66.90/69.31/62.07
	AUC	**1**/**1**/**1**	0.9809/0.9871/0.9676	**1**/**1**/**1**	0.9676/0.9712/0.9596	**0.9987**/**0.9962**/**0.9846**	0.7753/0.7698/0.6779
HD vs. HC	Sen (%)	**100**/**100**/**100**	93.28/91.37/93.42	**100**/**100**/**100**	85.89/89.16/87.45	**99.83**/**100**/**100**	78.84/76.27/89.42
	Spec (%)	**100**/**100**/**100**	81.14/85.05/89.27	**99.79**/**100**/**100**	81.34/77.68/77.24	**100**/**99.39**/**91.67**	67.82/61.72/62.52
	Acc (%)	**100**/**100**/**100**	87.04/88.06/91.11	**99.91**/**100**/**100**	82.31/81.39/77.78	**99.91**/**99.72**/**95.56**	61.76/67.78/61.11
	AUC	**1**/**1**/**1**	0.9431/0.9366/0.9688	**1**/**1**/**1**	0.8904/0.9034/0.9031	**1**/**1**/**1**	0.7186/0.7898/0.7969
PD vs. HC	Sen (%)	**99.13**/**97.47**/**100**	88.48/89.34/91.98	**92.86**/**91.09**/**89.70**	83.56/71.14/91.67	**94.46**/**89.96**/**60.69**	69.61/90.40/78.97
	Spec (%)	**94.53**/**96.42**/**96.47**	84.74/89.09/86.32	**91.39**/**91.16**/**94.62**	80.85/83.56/67.10	**92.33**/**95.07**/**76.99**	62.60/55.48/54.31
	Acc (%)	**96.45**/**96.77**/**98.06**	86.24/88.06/88.39	**91.29**/**90.97**/**90.32**	81.08/72.26/67.74	**92.47**/**91.29**/**61.94**	60.11/53.87/55.48
	AUC	**0.9992**/**0.9969**/**0.9967**	0.9222/0.9481/0.9450	**0.9794**/**0.9659**/**0.9679**	0.8907/0.8294/0.8954	**0.9957**/**0.9795**/**0.8763**	0.6908/0.6507/0.7150

Note: **bold and underlined** classification results were selected as the best based on Youden’s index.

**Table 5:** Summary results of all two-class classification states (among the NDD and all NDD in one group with HC group) using PCA.

Classification Task	Evaluation Parameter	Proposed Method					
		CWT + PCA (10 s/30 s/60 s)		STFT + PCA (10 s/30 s/60 s)		WSST + PCA (10 s/30 s/60 s)	
		LOOCV	*k*-Fold CV (*k* = 5)	LOOCV	*k*-Fold CV (*k* = 5)	LOOCV	*k*-Fold CV (*k* = 5)
ALS vs. HD	Sen (%)	97.95/96.83/100	**97.99**/**98.46**/**100**	96.93/93.43/92.19	99.01/96.18/95.79	96.70/87.60/90.32	97.88/82.21/89
	Spec (%)	98.67/96.08/93.46	**98.67**/**96.61**/**95.32**	98.16/98.96/94.06	97.10/97.54/93.06	98.49/91.54/91.26	96.30/95.38/88.49
	Acc (%)	98.38/96.36/95.76	**98.38**/**97.27**/**96.97**	97.68/96.67/93.33	97.78/96.97/93.33	97.78/90/90.91	96.87/86.67/85.45
	AUC	0.9831/0.9645/0.9673	**0.9953**/**0.9958**/**0.9800**	0.9755/0.9620/0.9312	0.9876/0.9851/0.9792	0.9760/0.8957/0.9079	0.9934/0.9687/0.9419
PD vs. ALS	Sen (%)	99.78/99.34/98.68	**99.78**/**99.35**/**98.75**	99.78/99.34/98.68	99.78/99.35/98.75	**99.78**/**98.04**/**94.94**	99.78/98.75/97.50
	Spec (%)	100/100/100	**100**/**100**/**100**	100/100/100	100/100/100	**100**/**100**/**100**	100/100/100
	Acc (%)	99.88/99.64/99.29	**99.88**/**99.64**/**99.29**	99.88/99.64/99.29	99.88/99.64/99.29	**99.88**/**99.64**/**97.14**	99.88/99.29/98.57
	AUC	0.9989/0.9967/0.9934	**1**/**1**/**0.9923**	0.9989/0.9967/0.9934	1/1/0.9923/0.9923	**0.9989**/**0.9902**/**0.9747**	0.9987/0.9962/0.9923
HD vs. PD	Sen (%)	98.82/**96.14**/94.17	**98.52**/95.28/**96.23**	94.93/93.30/96.08	94.61/94.78/94.85	94.21/89.16/86.60	89.79/93.43/96.80
	Spec (%)	96.72/**99.30**/95.83	**97.82**/99.31/**97.42**	95.44/96.45/97.26	97.01/95.75/96.40	93.05/87.07/79.49	99.51/82.95/84.09
	Acc (%)	97.90/**97.43**/94.86	**98.19**/96.86/**96.57**	95.14/94.57/96.57	95.52/94.57/94.86	93.71/88.29/83.43	93.14/86/89.14
	AUC	0.9777/**0.9772**/0.9500	**0.9982**/0.9953/**0.9940**	0.9519/0.9488/0.9667	0.9912/0.9926/0.9953	0.9363/0.8812/0.8304	0.9979/0.9540/0.9767
NDD vs. HC	Sen (%)	**99.58**/98.96/**97.94**	100/**99.38**/97.58	96.77/95.91/96.69	98.06/96.60/97.15	93.85/98.49/93.64	95.30/93.91/100
	Spec (%)	**95.76**/96.88/**97.40**	95.29/**96.47**/97.39	93.52/92.72/92.31	92.09/93.73/94.29	91.51/86.44/77.38	99.29/88.54/72.83
	Acc (%)	**98.59**/98.44/**97.81**	98.75/**98.59**/97.50	95.99/95.16//95.63	96.41/95.63/95.94	93.33/95.16/89.38	96.09/90.78/89.38
	AUC	**0.9767**/0.9792/**0.9767**	0.9991/**0.9959**/0.9689	0.9515/0.9431/0.9450	0.9844/0.9847/0.9742	0.9268/0.9246/0.8551	0.9978/0.9938/0.9943

Note: **bold and underlined** classification results were selected as the best based on Youden’s index.

**Table 6:** Comparison results of all two-class classification states between PCA and non-PCA (among the NDD and all NDD in one group with HC group) using *k*-fold CV (*k* = 5).

Classification Task	Evaluation Parameter	Proposed Method					
		CWT + PCA (10 s/30 s/60 s)		STFT + PCA (10 s/30 s/60 s)		WSST + PCA (10 s/30 s/60 s)	
		LOOCV	*k*-Fold CV (*k* = 5)	LOOCV	*k*-Fold CV (*k* = 5)	LOOCV	*k*-Fold CV (*k* = 5)
ALS vs. HD	Sen (%)	**97.99**/**98.46**/**100**	81.75/88.74/92.62	**99.01**/**96.18**/**95.79**	86.74/83.68/82.84	**97.88**/**82.21**/**89**	48.35/55.47/55.68
	Spec (%)	**98.67**/**96.61**/**95.32**	95.82/93.63/96.09	**97.10**/**97.54**/**93.06**	87.75/91.06/92.05	**96.30**/**95.38**/**88.49**	92.85/85.99/83.86
	Acc (%)	**98.38**/**97.27**/**96.97**	88.89/90.61/94.55	**97.78**/**96.97**/**93.33**	86.97/86.67/83.64	**96.87**/**86.67**/**85.45**	56.56/52.12/61.82
	AUC	**0.9953**/**0.9958**/**0.9800**	0.9584/0.9551/0.9788	**0.9876**/**0.9851**/**0.9792**	0.9410/0.9510/0.9519	**0.9934**/**0.9687**/**0.9419**	0.6849/0.6187/0.7612
PD vs. ALS	Sen (%)	**99.78**/**99.35**/**98.75**	91.99/80.17/86.08	**99.78**/**99.35**/**98.75**	86.12/87.07/85.79	**99.78**/**98.75**/**97.50**	74.55/63.51/84.04
	Spec (%)	**100**/**100**/**100**	88.84/85.75/79.51	**100**/**100**/**100**	83.91/74.38/76.07	**100**/**100**/**100**	63.60/86.06/69.66
	Acc (%)	**99.88**/**99.64**/**99.29**	90.36/81.43/80	**99.88**/**99.64**/**99.29**	84.17/77.50/77.14	**99.88**/**99.29**/**98.57**	64.40/64.64/57.14
	AUC	**1**/**1**/**0.9923**	0.9513/0.8835/0.8195	**1**/**1**/**0.9923**/**0.9923**	0.9315/0.8892/0.8938	**0.9987**/**0.9962**/**0.9923**	0.7588/0.6604/0.6641
HD vs. PD	Sen (%)	**98.52**/**95.28**/**96.23**	84.34/79.74/86.86	**94.61**/**94.78**/**94.85**	72.76/76.98/75.95	**89.79**/**93.43**/**96.80**	72.56/63.81/79.10
	Spec (%)	**97.82**/**99.31**/**97.42**	77.75/78.58/75.16	**97.01**/**95.75**/**96.40**	71.33/70.22/66.98	**99.51**/**82.95**/**84.09**	54.34/59.58/63.71
	Acc (%)	**98.19**/**96.86**/**96.57**	80.29/77.14/78.86	**95.52**/**94.57**/**94.86**	70.48/72.57/69.71	**93.14**/**86**/**89.14**	56.48/59.71/61.71
	AUC	**0.9982**/**0.9953**/**0.9940**	0.8869/0.8692/0.8457	**0.9912**/**0.9926**/**0.9953**	0.7580/0.7922/0.7817	**0.9979**/**0.9540**/**0.9767**	0.6515/0.5985/0.6963
NDD vs. HC	Sen (%)	**100**/**99.38**/**97.58**	90.57/94.41/88.94	**98.06**/**96.60**/**97.15**	89.69/89.21/90.01	**95.30**/**93.91**/**100**	83.12/89.43/83.38
	Spec (%)	**95.29**/**96.47**/**97.39**	77.63/74.75/83.47	**92.09**/**93.73**/**94.29**	65.08/58.69/66.96	**99.29**/**88.54**/**72.83**	46.99/38.03/70.67
	Acc (%)	**98.75**/**98.59**/**97.50**	87.19/87.97/84.38	**96.41**/**95.63**/**95.94**	82.71/78.28/74.38	**96.09**/**90.78**/**89.38**	65.99/48.91/69.06
	AUC	**0.9991**/**0.9959**/**0.9689**	0.8938/0.9308/0.8634	**0.9844**/**0.9847**/**0.9742**	0.8478/0.8280/0.8909	**0.9978**/**0.9938**/**0.9943**	0.6424/0.7777/0.7500

Note: **bold and underlined** classification results were selected as the best based on Youden’s index.

**Table 7:** Summary results of all multi-class classification states using PCA for 10-s time window.

Classification Class	Evaluation Parameter	Proposed Method					
		CWT + PCA		STFT + PCA		WSST + PCA	
		LOOCV	*k*-Fold CV (*k* = 5)	LOOCV	*k*-Fold CV (*k* = 5)	LOOCV	*k*-Fold CV (*k* = 5)
HC	Sen (%)	**98.75**	97.71	92.50	94.79	90	87.71
	Spec (%)	**99.03**	98.89	97.92	97.64	96.18	97.71
	Acc (%)	**98.96**	98.59	96.56	96.93	94.64	95.21
	AUC	**0.9889**	0.9830	0.9521	0.9622	0.9309	0.9271
ALS	Sen (%)	**98.21**	97.95	97.44	97.69	97.18	95.90
	Spec (%)	**99.35**	99.80	99.35	98.76	98.95	99.02
	Acc (%)	**99.11**	99.43	98.96	98.54	98.59	98.39
	AUC	**0.9878**	0.9888	0.9839	0.9823	0.9807	0.9746
HD	Sen (%)	97	**97**	95	93.83	89.83	89.33
	Spec (%)	98.86	**99.17**	96.52	96.74	96.89	98.11
	Acc (%)	98.28	**98.49**	96.04	95.83	94.69	95.36
	AUC	0.9793	**0.9808**	0.9576	0.9529	0.9336	0.9372
PD	Sen (%)	**95.33**	96.22	85.33	86.22	86.22	83.78
	Spec (%)	**98.64**	97.21	96.19	96.73	91.90	92.52
	Acc (%)	**97.86**	96.98	93.65	94.27	90.57	90.47
	AUC	**0.9699**	0.9808	0.9076	0.9148	0.8906	0.8815

Note: **bold and underlined** classification results were selected as the best based on Youden’s index.

**Table 8:** Summary results of all multi-class classification states using PCA for 30-s time window.

Classification Class	Evaluation Parameter	Proposed Method					
		CWT + PCA		STFT + PCA		WSST + PCA	
		LOOCV	*k*-Fold CV (*k* = 5)	LOOCV	*k*-Fold CV (*k* = 5)	LOOCV	*k*-Fold CV (*k* = 5)
HC	Sen (%)	96.25	**97.50**	89.38	92.50	86.25	91.88
	Spec (%)	99.17	**98.54**	97.92	97.71	96.88	96.46
	Acc (%)	98.44	**98.28**	95.78	96.41	94.22	95.31
	AUC	0.9771	**0.9802**	0.9365	0.9510	0.9156	0.9417
ALS	Sen (%)	**96.92**	95.38	**96.92**	96.92	93.08	98.46
	Spec (%)	**98.63**	98.82	**98.63**	98.24	95.88	91.76
	Acc (%)	**98.28**	98.13	**98.28**	97.97	95.31	93.13
	AUC	**0.9778**	0.9710	**0.9778**	0.9758	0.9448	0.9511
HD	Sen (%)	93.50	93.50	**94**	94.50	76.50	84.50
	Spec (%)	97.27	97.73	**97.50**	95	95.68	95
	Acc (%)	96.09	96.41	**96.41**	94.84	89.69	91.72
	AUC	0.9539	0.9561	**0.9575**	0.9475	0.8609	0.8975
PD	Sen (%)	**92.67**	94	88	84.67	83.33	76.67
	Spec (%)	**98.16**	94.69	93.88	96.53	90.20	93.06
	Acc (%)	**96.88**	94.53	92.50	93.75	88.59	89.22
	AUC	**0.9541**	0.9435	0.9094	0.9060	0.8677	0.8486

Note: **bold and underlined** classification results were selected as the best based on Youden’s index.

**Table 9:** Summary results of all multi-class classification states using PCA for 60-s time window.

Classification Class	Evaluation Parameter	Proposed Method					
		CWT + PCA		STFT + PCA		WSST + PCA	
		LOOCV	*k*-Fold CV (*k* = 5)	LOOCV	*k*-Fold CV (*k* = 5)	LOOCV	*k*-Fold CV (*k* = 5)
HC	Sen (%)	95	**98.75**	92.50	97.50	78.75	76.25
	Spec (%)	99.17	**97.92**	97.50	98.04	92.92	88.75
	Acc (%)	98.13	**98.13**	96.25	94.38	89.38	85.63
	AUC	0.9708	**0.9833**	0.9500	0.9542	0.8583	0.8250
ALS	Sen (%)	**92.31**	93.85	90.77	86.15	78.46	75.38
	Spec (%)	**98.04**	96.86	97.25	98.82	98.82	90.20
	Acc (%)	**96.88**	96.25	95.94	96.25	94.69	87.19
	AUC	**0.9517**	0.9535	0.9401	0.9249	0.8864	0.8279
HD	Sen (%)	**98**	95	95	94	75	92
	Spec (%)	**93.64**	95.45	94.09	95	91.36	87.27
	Acc (%)	**95**	95.31	94.38	94.69	86.25	88.75
	AUC	**0.9582**	0.9523	0.9455	0.9450	0.8318	0.8964
PD	Sen (%)	89.33	**89.33**	85.33	82.67	68	57.33
	Spec (%)	97.55	**97.96**	93.88	95.51	83.27	88.98
	Acc (%)	95.63	**95.94**	91.88	92.50	79.69	81.56
	AUC	0.9344	**0.9365**	0.8961	0.8909	0.7563	0.7316

Note: **bold and underlined** classification results were selected as the best based on Youden’s index.

**Table 10:** A comparison of the performance of the proposed method using LOOCV.

Studies	Evaluation Parameter	Classification Task			
		ALS vs. HC	HD vs. HC	PD vs. HC	NDD vs. HC
Zeng et al. [22]	Sen (%)	92.31	85	87.50	-
	Spec (%)	87.50	81.25	86.67	-
	Acc (%)	89.66	87.10	87.10	-
Zhao et al. [23]	Acc (%)	97.43	94.96	97.33	96.42
Ren et al. [64]	AUC	0.8980	0.8810	0.9010	-
Pham, T.D. [63]	Sen (%)	100	100	100	-
	Spec (%)	100	100	100	-
	Acc (%)	100	100	100	-
	AUC	1	1	1	-
The Proposed Method	Sen (%)	100	100	99.08	98.96
	Spec (%)	100	100	95.97	96.88
	Acc (%)	100	100	97.42	98.44
	AUC	1	1	0.9752	0.9792

## Data Availability

Not applicable.
